# Novel proteolytic post-translational modification in voltage-gated potassium channel KCNQ2

**DOI:** 10.1038/s41598-026-42444-9

**Published:** 2026-03-04

**Authors:** Yuichi Kimura, Hidehiko Uchiyama, Koji Masuda, Shinichi Hirose

**Affiliations:** 1 Department of Animal Science, Tokyo University of Agriculture, Kanagawa, Japan; 2 General Medical Research Center, School of Medicine, Fukuoka University, Fukuoka, Japan

**Keywords:** Voltage-gated potassium channel, KCNQ2, Kv7.2, Post-translational modification (PTM), Proteolytic cleavage, Epilepsy, Diseases, Neurology, Neuroscience

## Abstract

KCNQ2 is a member of the voltage-gated potassium (Kv) channel family and regulates neuronal activity through potassium ion efflux. Pathogenic variants of KCNQ2 induce aberrant neuronal activity and cause two types of epilepsy: self-limited familial neonatal epilepsy (SLFNE) and developmental and epileptic encephalopathies (DEE). However, the molecular mechanism by which these pathogenic variants influence KCNQ2 expression remains unclear. Here, we show N-terminal and C-terminal fragments derived from mouse KCNQ2 (KCNQ2^S−N^ and KCNQ2^S−C^, respectively), whose amounts differed significantly across variants compared with wild type, whereas those of full-length KCNQ2 (KCNQ2^F^) remained unchanged. Of particular interest, two variants at the same codon, Y284C and Y284D, which are associated with distinct clinical phenotypes—self-limited familial neonatal epilepsy (SLFNE) and developmental and epileptic encephalopathy (DEE), respectively—exerted opposite effects on the fragment: Y284C increased the amounts of both KCNQ2 fragments, whereas Y284D decreased it compared with the wild type. As both KCNQ2^S−N^ and KCNQ2^S−C^ were localized in the plasma membrane, both fragments were suggested to be post-translational products resulting from a cleavage of full-length KCNQ2. This novel post-translational cleavage was observed in neuronal cells and appears to be evolutionarily conserved. Although the role of this post-translational modification in epilepsy remains unknown, it may be elucidated through future studies.

**Supplementary Information:**

The online version contains supplementary material available at 10.1038/s41598-026-42444-9.

## Introduction

Voltage-gated potassium (Kv) channels are essential for maintaining the resting membrane potential and regulating neuronal excitability^1^. The subunit of these ion channels consists of six transmembrane segments S1 to S6 that are classified into 12 subfamilies^2–4^. The human *KCNQ2* (*hKCNQ2*) gene encodes Kv7.2, a member of the Kv7 channel family. Kv7.2 forms homo- or hetero-tetramers with Kv7.3, encoded by *hKCNQ3*, a known paralog of *hKCNQ2*^5,6^.

As per previous studies, pathogenic variants in *hKCNQ2* are associated with two distinct epilepsy syndromes—self-limited familial neonatal epilepsy (SLFNE) and developmental and epileptic encephalopathy (DEE)^7–10^. Both conditions typically manifest within the first few days after birth; SLFNE usually resolves within weeks with a good prognosis, whereas DEE represents an intractable form of epilepsy accompanied by developmental delay^6^. Diverse pathogenic variants in *hKCNQ2*, including frameshift, insertion, deletion, and single-nucleotide polymorphisms (SNPs), have been identified in affected individuals^11,12^. Most *KCNQ2* variants are associated with loss-of-function rather than gain-of-function effects^4,13–15^.

Several studies have reported that pathogenic variants of *KCNQ2* alter protein expression levels and subcellular localization in neurons^11,16–18^. However, the mechanisms by which these mutations affect KCNQ2 protein processing and expression remain largely unknown. To explore this issue, we analyzed the expression of mouse KCNQ2 (mKCNQ2) carrying several pathogenic variants previously identified in either self-limited familial neonatal epilepsy (SLFNE) or developmental and epileptic encephalopathy (DEE), including T274M, Y284C, Y284D, G290D, and A306T^19–22^.

In this study, we discovered a novel post-translational modification of KCNQ2—a proteolytic cleavage that generates fragments—and found that the proportion of this truncated form varied depending on the mutation.

## Results

### Different expression patterns of mKCNQ2 across genotypes

**Fig. 1:**
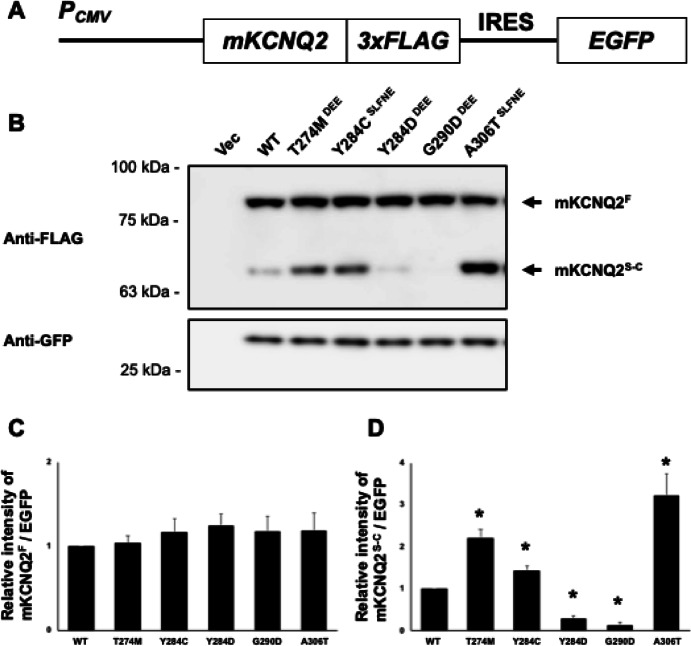
Amount of C-terminal fragment of mKCNQ2 differs significantly among genetic variants. To analyze mKCNQ2 protein expression, we generated plasmids encoding each variant of *mKcnq2* fused to a 3×FLAG- tagged carboxyl-terminal and independently expressing EGFP via an IRES sequence (**A**). Plasmids were transfected into Neuro2A cells. mKCNQ2 and GFP proteins were immunoblotted with anti-FLAG and anti-GFP antibodies, respectively (**B**). The intensities of mKCNQ2-FLAG were measured and normalized to GFP levels, and compared with WT. Data are presented as the mean ± SEM (*n* = 5). *p < 0.05, determined using Kruskal–Wallis test followed by Steel’s multiple compression test (**C** and** D**). T274M, Y284D, and G290D are pathogenic variants of the DEE, whereas Y284C and A306T are pathogenic variants of the SLFNE. Original blots are presented in Supplementary Fig. 2.

To investigate the basic gene expression profile of mouse *KCNQ2* (*mKcnq2*) using mouse neuronal blastoma cell line, Neuro2A cell, we focused on five genotypes previously reported as pathogenic mutations: Thr274Met (T274M), Tyr284Asp (Y284D), and Gly290Asp (G290D), associated with DEE; and Tyr284Cys (Y284C) and Ala306Thr (A306T), associated with SLFNE^19–22^. We constructed carboxyl-terminal 3xFLAG (“DYKDDDDK”)-tagged *mKcnq2* plasmids harboring each variant (Fig. 1A) and examined mKCNQ2 protein expression using western blotting with an anti-FLAG antibody (Fig. 1B). Full-length mKCNQ2 (mKCNQ2^F^) protein levels were not significantly different among the genotypes (Fig. 1B and C). However, we observed an additional low-molecular-weight band, which appeared to be missing the N-terminus, and hence named it C-terminal fragment of mKCNQ2 (mKCNQ2^S−C^). Quantitative analysis showed that mKCNQ2^S−C^ amount was significantly increased in the T274M, Y284C, and A306T variants but decreased in Y284D and G290D compared with that in WT (Fig. 1B and D). Notably, Y284C and Y284D, which differ by a single amino acid substitution, exhibited opposite amount trends. Together, these results suggest that the five pathogenic SNPs affect the amounts of mKCNQ2 fragments, as compared to WT.

### mKCNQ2 may undergo cleavage via post-translational modification

**Fig. 2:**
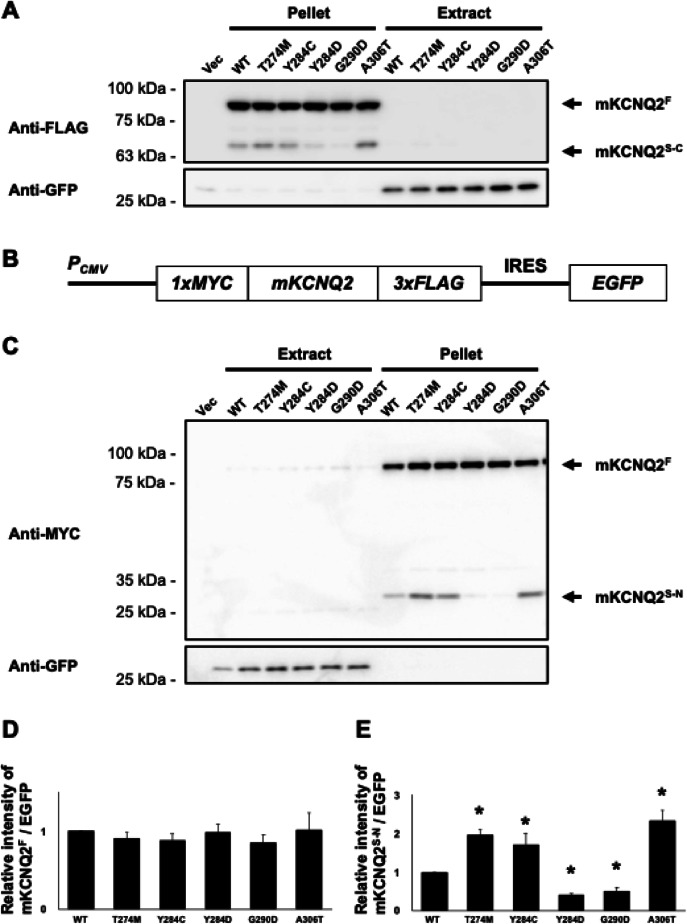
N-terminal and C-terminal fragments of mKCNQ2 detected in the pellet fraction. Each *mKcnq2* plasmid was transfected into Neuro2A cells. Whole-cell extracts were separated into soluble (extract) and pellet fractions. mKCNQ2 samples were immunoblotted with anti-FLAG and anti-GFP antibodies (**A**). To determine amino-terminal mKCNQ2 protein expression, we generated encoding *mKcnq2* variants fused with an N-terminal 1×Myc tag and a C-terminal 3×FLAG tag (**B**). Separated cell extracts were analyzed using immunoblotting with anti-Myc and anti-GFP antibodies (**C**). The intensities of Myc-mKCNQ2 were measured and normalized to GFP levels, and compared with WT. Data are presented as the mean ± SEM (*n* = 5). *p < 0.05, determined using Kruskal–Wallis test followed by Steel’s multiple compression test (**D** and** E**). Original blots are presented in Supplementary Figs. 3 and 4.

While mKCNQ2^F^ is known to localize in the cell membrane, the subcellular localization of mKCNQ2^S−C^ remains unclear. To address this, we separated cells into cytoplasmic and pellet fractions and performed western blotting. Both mKCNQ2^F^ and mKCNQ2^S−C^ were detected in the pellet fraction (Fig. 2A), suggesting their membrane association. The mechanism underlying mKCNQ2^S−C^ synthesis remained unclear. We therefore hypothesized that mKCNQ2^S−C^ arises either from aberrant translational initiation or from protein cleavage. To examine this hypothesis, we generated *mKcnq2* plasmids bearing an amino-terminal 1xMYC tag and a carboxyl–terminal 3xFLAG tag harboring each genotype (Fig. 2B) and the full-length amino acid sequence is shown in Supplementary Fig. 1. Western blotting with anti-Myc antibody showed two bands that yielded similar results with anti-FLAG (Fig. 2C, D and E); we named it the N-terminal fragment of mKCNQ2 (mKCNQ2^S−N^). This result supports the hypothesis that the fragments result from protein cleavage rather than aberrant translation initiation.

Based on the possibility that mKCNQ2 undergoes proteolytic cleavage, we investigated the potential cleavage site. Western blotting using anti-Myc (N-terminal) and anti-FLAG (C-terminal) antibodies detected two distinct fragments of approximately 30 kDa and 65 kDa, respectively (Fig. 2A and C). The 65 kDa C-terminal fragment was designated mKCNQ2^S−C^, whereas the 30 kDa N-terminal fragment mKCNQ2^S−N^ corresponded to the remaining portion of the full-length protein, approximetry 90 kDa. The combined molecular weights of these two fragments were roughly equivalent to that of mKCNQ2^F^, indicating that both fragments represent a product generated through post-translational cleavage of mKCNQ2^F^.

**Fig. 3:**
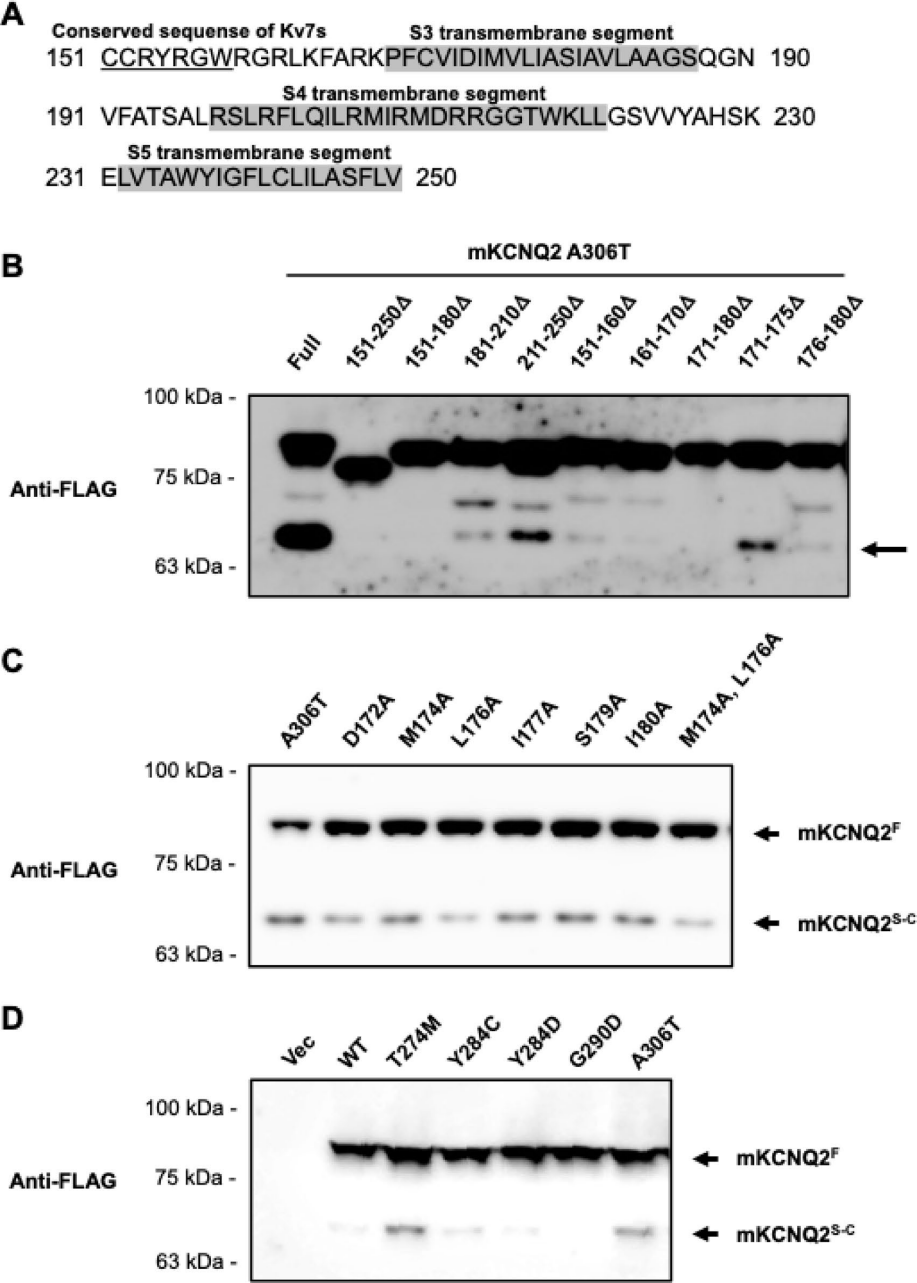
Analysis of the amino acid region required for fragment generation. Amino acid residues 151 to 250 of mKCNQ2 are shown (**A**). To determine the region required for fragment generation, we generated deletion mutants lacking specific amino acids based on the A306T variant of mKCNQ2. These plasmids were transfected into Neuro2A cells and mKCNQ2 protein expression was assessed via immunoblotting with anti-FLAG antibody (**B**). To observe the effects of protease, any amino acid residue was replaced with alanine of mKCNQ2. The plasmids were then transfected into Neuro2A cells, and mKCNQ2 protein expression was assessed via immunoblotting with anti-FLAG antibody (**C**). To analyze the amounts of fragments, we added protease inhibitor 30 min before transfection in Neuro2A cells. mKCNQ2 samples were immunoblotted with anti-FLAG antibodies (**D**). Original blots are presented in Supplementary Figs. 5, 6, and 7.

Based on these findings, we hypothesized that the cleavage site was located within a 100–amino acid region spanning residues 151–250, which encompasses the S3 to S5 transmembrane segments (Fig. 3A). To test this, we constructed *mKcnq2* plasmids carrying deletions within this region on the A306T background, which exhibits high amount of mKCNQ2^S−C^. Among these constructs, deletion of residues 171–180 abolished the mKCNQ2^S−C^ band while preserving the band of the full-length form (mKCNQ2^F^) (Fig. 3B). This result suggested that the cleavage site is likely located around residues 171–180. Supporting this, smaller deletions of residues 171–175 or 176–180 still produced detectable mKCNQ2^S−C^ (Fig. 3B).

In silico analysis using peptide cutter software of Expasy, which is operated by the Swiss Institute of Bioinformatics (https://web.expasy.org/peptide_cutter/), predicted that the 10 amino acids region (171–180) included cleavage sites for Asp-N endopeptidase and chymotrypsin. To experimentally verify this prediction, we performed alanine-scanning mutagenesis, a method in which amino acid residues within a target region are systematically replaced with alanine. Because alanine lacks bulky or charged side chains, such substitutions often disrupt protease recognition motifs and render the region resistant to enzymatic cleavage. Using this approach, we generated *mKcnq2* plasmids in which the residues within the predicted cleavage region (171–180) were substituted with alanine. However, mKCNQ2^S−C^ was still detected in all mutant constructs (Fig. 3C). Finally, addition of protease inhibitor for serine, cysteine, and aspartic proteases and aminopeptidases did not abolish cleavage of mKCNQ2 (Fig. 3D). Taken together, these results suggest that both mKCNQ2^F^ and the fragments are expressed on the membrane, and that mKCNQ2 may be cleaved via a novel post-translational modification mechanism.

### mKCNQ3 expression pattern differs from that of mKCNQ2

**Fig. 4:**
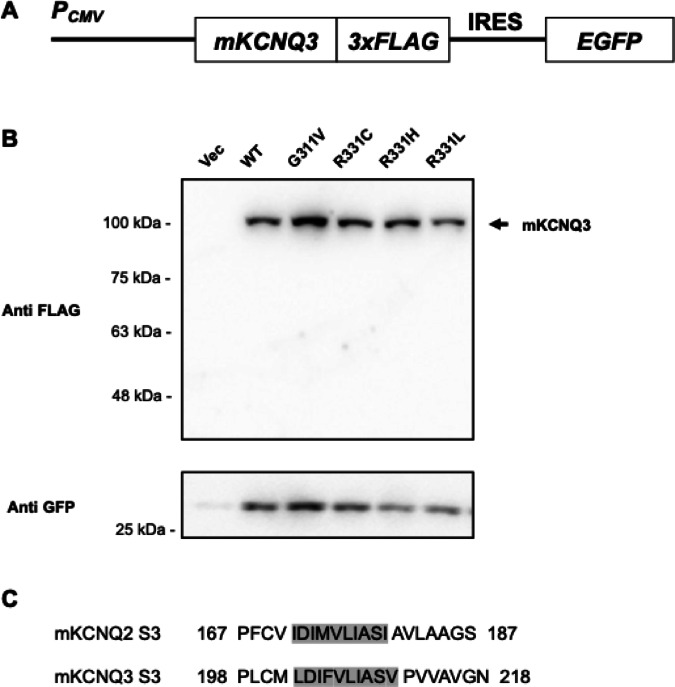
Cleaved fragments do not appear in mKCNQ3. To analyze mKCNQ3 protein expression, we generated each plasmid encoding C-terminal 3×FLAG-tagged *mKcnq3* variants with independent EGFP expression via an IRES sequence (**A**). Plasmids were transfected into Neuro2A cells. mKCNQ3 and GFP proteins were immunoblotted with anti-FLAG and anti-GFP antibodies (**B**). Comparison of amino acid sequences of the S3 between mKCNQ2 and mKCNQ3. Gray highlighted area represents predicted cleavage region (**C**). Original blots are presented in Supplementary Fig. 8.

hKCNQ3 forms both homo- and/or hetero-tetramers with hKCNQ2 and has been implicated in conditions including SLFNE and neurocognitive deficits^4,7,23–25^. However, as with *hKCNQ2*, it remains unclear how specific genetic variants in *hKCNQ3* gene contribute to disease pathogenesis. Given that *hKCNQ3* is a paralog of *hKCNQ2*, we hypothesized that mKCNQ3 may also get cleaved, similar to mKCNQ2. To verify this, we constructed carboxyl–terminally 3xFLAG-tagged *mKcnq3* plasmids encoding genotypes of WT as well as the variants G311V, R331C, R331H, and R331L (Fig. 4A). Notably, western blot analysis revealed that only full-length mKCNQ3 was detected (Fig. 4B), with no evidence of a cleaved protein. The amino acid sequence homology between the S3 of mKCNQ2 and mKCNQ3 was 57%, specifically 70% for the predicted cleavage site (Fig. 4C). These results suggest that the regulatory mechanism of gene expression differs between mKCNQ2 and mKCNQ3.

### KCNQ2 cleavage may be regulated by post-translational modification, similar to that for mKCNQ2

**Fig. 5:**
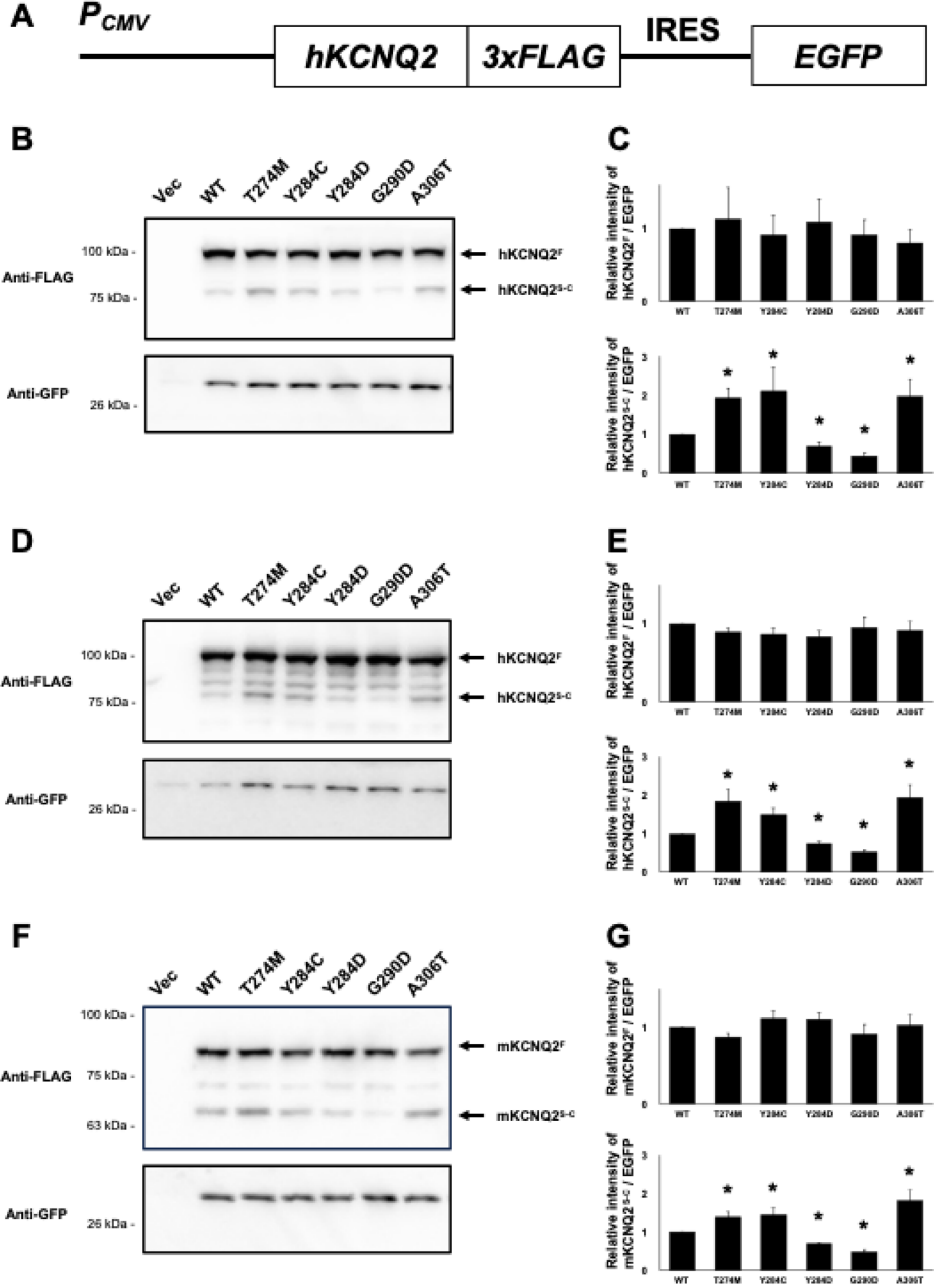
Post-translational cleavage is evolutionarily conserved. To analyze hKCNQ2 protein expression, we generated plasmids encoding each variant of *hKcnq2* fused to a 3×FLAG-tagged carboxyl-terminal and independently expressing EGFP via an IRES sequence (**A**). Plasmids were transfected into Neuro2A and SH-SY5Y cells. hKCNQ2 and GFP proteins were immunoblotted with anti-FLAG and anti-GFP antibodies, and quantified (**B**,** C**,** D**, and** E**). *mKcnq2* plasmids were also transfected in HEK293T cells, and mKCNQ2 and GFP proteins were detected using immunoblotting with anti-FLAG and anti-GFP antibodies, and quantified (F and G). The intensities of each KCNQ2-FLAG were measured and normalized to GFP levels, and compared with WT. All data are presented as mean ± SEM (*n* = 5). *p < 0.05, determined using Kruskal–Wallis test followed by Steel’s multiple compression test. Original blots are presented in Supplementary Figs. 9, 10, and 11.

While our analyses focused on mKCNQ2, whether human KCNQ2 is also subject to proteolytic cleavage remained unclear. Few studies have focused on the molecular mechanisms regulating KCNQ2 gene expression. To determine whether protein cleavage of mKCNQ2 represents a mouse-specific post-translational control or an evolutionarily conserved mechanism, we constructed carboxyl-terminal 3xFLAG-tagged human KCNQ2 expression plasmids and examined KCNQ2 expression pattern (Fig. 5A). Western blot analysis showed the presence of both KCNQ2^F^ and KCNQ2^S−C^, and the expression patterns including statistical analysis results of individual variants mirrored those obtained for mKCNQ2 (Fig. 5B and C), indicating that the mechanism of KCNQ2 cleavage is conserved between human and mouse. To further investigate whether the cleavage is specific to cell type or species, we expressed hKCNQ2 in the human neuroblastoma cell line SH-SY5Y and mKCNQ2 in HEK293T cells. In both systems, we could detect cleaved KCNQ2 bands, and detection patterns including quantitative analysis were equivalent to those presented in Fig. 1 results (Fig. 5D, E, F and G).

These findings suggest that KCNQ2 cleavage is an evolutionarily conserved post-translational mechanism that occurs not only in heterologous expression systems but also in neuronal cells. This indicates that proteolytic processing of KCNQ2 is a physiologically relevant event, rather than an artifact limited to HEK293T cells.

## Discussion

Pathogenic variants in KCNQ2 are associated with two clinically distinct epileptic disorders—self-limited familial neonatal epilepsy (SLFNE) and developmental and epileptic encephalopathy (DEE)—yet the molecular basis underlying their phenotypic divergence remains poorly understood. While most previous studies have emphasized electrophysiological abnormalities caused by channel dysfunction, relatively little is known about how these variants influence KCNQ2 expression and post-translational regulation.

In line with earlier reports^11,26^, our study found no marked differences in total KCNQ2 mRNA or protein levels among the tested variants, indicating that endoplasmic-reticulum–associated degradation (ERAD) or calmodulin-dependent mechanisms are unlikely to explain the variant-dependent phenotypes. Instead, we identified previously uncharacterized fragments of KCNQ2, namely KCNQ2^S−N^ and KCNQ2^S−C^, that result from post-translational cleavage of the full-length protein (KCNQ2^F^). Although similar short bands have occasionally been observed in earlier studies^16^, their significance had not been addressed. Here, we demonstrate that the amounts of fragments vary depending on genotype, most notably between the Y284C and Y284D variants, which are associated with SLFNE and DEE, respectively.

Biochemical analysis revealed that KCNQ2^S−C^ corresponds to the C-terminal fragment generated by cleavage within the S3 of KCNQ2. The 171–180 amino acid region was found to be essential for the appearance of fragments, implying that this segment harbors a critical cleavage site. The S3 plays a central role in voltage sensing through its interaction with S2 and S4 segments^4,14,27,28^. In the resting state, residue D172 in S3 forms electrostatic interactions with R201 or R213 in S4, contributing to channel gating. Cleavage within this region could therefore alter conformational coupling between the voltage-sensing and pore domains, potentially modulating channel function. However, the impact of structural changes due to amino acid deletions could not be ruled out, necessitating further verification.

The proteolytic mechanism responsible for generating the fragments remains to be elucidated. The fact that neither alanine-scanning mutagenesis nor protease inhibitor treatment prevented fragment formation suggests the involvement of a noncanonical cleavage process. Given that the S3 resides within the transmembrane region, accessibility by conventional proteases is limited. One possible explanation is the participation of intramembrane-cleaving proteases (i-CLiPs), such as γ-secretase, site-2 protease (S2P), and rhomboid proteases^29–32^. These evolutionarily conserved enzymes mediate regulated intramembrane proteolysis, and some act on ion channel proteins, although their specific substrate sequences remain incompletely characterized^33–36^. Our observations are consistent with the possibility that an i-CLiP–like mechanism contributes to KCNQ2 cleavage.

The functional significance of the fragments remains speculative, but its conservation across species and occurrence in neuronal as well as non-neuronal cells suggests a physiologically relevant process. Cleavage may serve to regulate the stability, localization, or turnover of Kv7.2 channels under specific cellular conditions. The finding that the Y284C and Y284D variants—linked to opposite clinical phenotypes—induce inverse effects on the fragment amounts supports the notion that dysregulation of this post-translational process could influence disease pathogenesis. Whether differences in the fragment amounts directly alter channel conductance or subunit assembly remains to be clarified.

In summary, our study identifies a novel post-translational cleavage of KCNQ2 within the S3 and demonstrates that the relative amounts of the fragments vary among pathogenic variants. This cleavage process appears evolutionarily conserved and occurs in neuronal cells, implying a potential role in the physiological regulation of KCNQ2 function. Future investigations should aim to identify the responsible protease and elucidate how altered fragment formation contributes to epileptic phenotypes such as SLFNE and DEE.

## Methods

### Mouse *Kcnq2*, *Kcnq3*, and human *KCNQ2* gene cloning and plasmid preparation

Coding sequences of mouse *Kcnq2*, *Kcnq3*, and human *KCNQ2* were amplified via PCR using Mouse Brain QUICK-Clone cDNA and Human Fetal Brain QUICK-Clone cDNA as templates (Clontech, Palo Alto, CA, USA). The amplified *mKcnq2*, m*Kcnq3*, and *hKCNQ2* plasmids were constructed using the pIRES2-EGFP vector (Clontech, Palo Alto, CA, USA) and the In-Fusion HD Cloning Kit (Clontech, Palo Alto, CA, USA). Amino-terminal Myc and carboxyl-terminal FLAG tags were introduced using the same cloning strategy. Site-directed mutagenesis was performed using the KOD -Plus- Mutagenesis kit (Toyobo Co., Ltd., Osaka, Japan) to generate plasmids harboring each genetic variant.

### Cell culture, transfection, protein extraction, and cell treatments

Neuro2A and HEK293T cells were cultured in Dulbecco’s modified Eagle’s medium (DMEM, high glucose; Sigma-Aldrich, St. Louis, MO, USA) supplemented with 10% fetal bovine serum (FBS). SH-SY5Y cells were cultured in DMEM/Nutrient Mixture F-12 Ham (Sigma-Aldrich, St. Louis, MO, USA) supplemented with 10% FBS and MEM Non-essential Amino Acid Solution (Sigma-Aldrich, St. Louis, MO, USA). All cells were incubated at 37 °C in a humidified 5% CO_2_ atmosphere. Transfection of cells was performed using Lipofectamine 3000 (Thermo Fisher Scientific, Waltham, MA, USA) with 1 µg of plasmid DNA, according to the manufacturer’s instructions. Twenty-four hours post-transfection, cells were washed with PBS. Extract and pellet fractions were separated from the cultured cells using an extraction buffer (20 mM Tris-HCl, 15 mM NaCl, and 0.1% Triton X-100). Proteins were extracted by mixing the cell lysates with SDS sample buffer and sonicating the samples. Protease inhibitor cocktail (P1860; Sigma-Aldrich, St. Louis, MO, USA) was added to cells 30 min before transfection to prevent protein degradation.

Western blot analysis, antibodies, quantification, and statistical analysisFor Western blot analysis, the following antibodies were used: anti-FLAG M2 monoclonal antibody (F1804; Sigma-Aldrich, St. Louis, MO, USA), anti-GFP monoclonal antibody (1E4; Medical & Biological Laboratories Co., Ltd., Tokyo, Japan), and anti-Myc monoclonal antibody (My3; Medical & Biological Laboratories Co., Ltd., Tokyo, Japan). Phosphorylated proteins were separated using Phos-tag Acrylamide (Fuji Film Wako Pure Chemical Co., Tokyo, Japan) according to the manufacturer’s instructions. Protein detection and molecular weight analysis were performed using the FUSION SOLO.6 S.EDGE imaging system (Vilber Bio Imaging, Paris, France) with Immobilon Western Chemiluminescent Horseradish Peroxidase Substrate (Merck Millipore, Billerica, MA, USA). Signal intensities were quantified using the analysis software attached to the AI680 system. Statistical analyses were performed using BellCurve for Excel (Social Survey Research Information Co., Ltd., Tokyo, Japan).

## Supplementary Information

Below is the link to the electronic supplementary material.

Supplementary Material 1

## Data Availability

The data that support the findings of this study are available from the corresponding author upon reasonable request.

## References

[1] Wang, H. S. et al. KCNQ2 and KCNQ3 potassium channel subunits: molecular correlates of the M-channel. *Science* **282**, 1890–1893 (1998). 9836639 10.1126/science.282.5395.1890

[2] Gutman, G. A. et al. International union of pharmacology. XLI. Compendium of voltage-gated ion channels: Potassium channels. *Pharmacol. Rev.* **55**, 583–586 (2003). 14657415 10.1124/pr.55.4.9

[3] Gutman, G. A. et al. International union of pharmacology. LIII. Nomenclature and molecular relationships of voltage-gated potassium channels. *Pharmacol. Rev.* **57**, 473–508 (2005). 16382104 10.1124/pr.57.4.10

[4] Nappi, P. et al. Epileptic channelopathies caused by neuronal Kv7 (KCNQ) channel dysfunction. *Pflugers Arch.* **472**, 881–898 (2020). 32506321 10.1007/s00424-020-02404-2

[5] Ihara, Y. et al. Retigabine, a Kv7.2/Kv7.3-channel opener, attenuates drug-induced seizures in knock-in mice harboring Kcnq2 mutations. *PLoS One*. **11**, e0150095. 10.1371/journal.pone.0150095 (2016). 26910900 10.1371/journal.pone.0150095 PMC4766199

[6] Miceli, F. et al. A novel Kv7. 3 variant in the voltage-sensing S4 segment in a family with benign neonatal epilepsy: functional characterization and in vitro rescue by β-hydroxybutyrate. *Front. Physiol.* **11**, 1040. 10.3389/fphys.2020.01040 (2020). 33013448 10.3389/fphys.2020.01040 PMC7498716

[7] Schroeder, B. C., Kubisch, C., Stein, V. & Jentsch, T. J. Moderate loss of function of cyclic-AMP-modulated KCNQ2/KCNQ3 K+ channels causes epilepsy. *Nature* **396**, 687–690 (1998). 9872318 10.1038/25367

[8] Kato, M. et al. Clinical spectrum of early onset epileptic encephalopathies caused by KCNQ2 mutation. *Epilepsia* **54**, 1282–1287 (2013). 23621294 10.1111/epi.12200

[9] Vanoye, C. G. et al. High-throughput evaluation of epilepsy-associated KCNQ2 variants reveals functional and pharmacological heterogeneity. *JCI insight*. **7**, e156314. 10.1172/jci.insight.156314 (2022). 35104249 10.1172/jci.insight.156314 PMC8983144

[10] Ishii, A. et al. A *de novo* KCNQ2 mutation detected in non-familial benign neonatal convulsions. *Brain Dev.* **31**, 27–33 (2009). 18640800 10.1016/j.braindev.2008.05.010

[11] Orhan, G. et al. Dominant-negative effects of KCNQ2 mutations are associated with epileptic encephalopathy. *Ann. Neurol.* **75**, 382–394 (2014). 24318194 10.1002/ana.24080

[12] Goto, A. et al. Characteristics of KCNQ 2 variants causing either benign neonatal epilepsy or developmental and epileptic encephalopathy. *Epilepsia* **60**, 1870–1880 (2019). 31418850 10.1111/epi.16314 PMC11812603

[13] Devaux, J. et al. A Kv7. 2 mutation associated with early onset epileptic encephalopathy with suppression-burst enhances Kv7/M channel activity. *Epilepsia* **57** (93), e87. 10.1111/epi.13366 (2016). 27030113 10.1111/epi.13366

[14] Miceli, F. et al. Early-onset epileptic encephalopathy caused by gain-of-function mutations in the voltage sensor of Kv7. 2 and Kv7. 3 potassium channel subunits. *J. Neurosci.* **35**, 3782–3793 (2015). 25740509 10.1523/JNEUROSCI.4423-14.2015 PMC6605567

[15] Mulkey, S. B. et al. Neonatal nonepileptic myoclonus is a prominent clinical feature of KCNQ 2 gain‐of‐function variants R201C and R201H. *Epilepsia* **58**, 436–445 (2017). 28139826 10.1111/epi.13676 PMC5339037

[16] Tran, B., Ji, Z. G., Xu, M., Tsuchida, T. N. & Cooper, E. C. Two KCNQ2 encephalopathy variants in the calmodulin-binding helix A exhibit dominant-negative effects and altered PIP2 interaction. *Front. Physiol.* **11**, 1144 (2020). 33041849 10.3389/fphys.2020.571813 PMC7518097

[17] Abidi, A. et al. A recurrent KCNQ2 pore mutation causing early onset epileptic encephalopathy has a moderate effect on M current but alters subcellular localization of Kv7 channels. *Neurobiol. Dis.* **80**, 80–92 (2015). 26007637 10.1016/j.nbd.2015.04.017

[18] Abreo, T. J. et al. Plural molecular and cellular mechanisms of pore domain KCNQ2 encephalopathy. *Elife* **13**, RP91204. 10.7554/eLife.91204 (2025). 39761077 10.7554/eLife.91204 PMC11703504

[19] Hortigüela, M. et al. Clinical and genetic features of 13 Spanish patients with KCNQ2 mutations. *J. Hum. Genet.* **62**, 185–189 (2017). 27535030 10.1038/jhg.2016.104

[20] Weckhuysen, S. et al. KCNQ2 encephalopathy: emerging phenotype of a neonatal epileptic encephalopathy. *Ann. Neurol.* **71**, 15–25 (2012). 22275249 10.1002/ana.22644

[21] Millichap, J. J. et al. KCNQ2 encephalopathy: Features, mutational hot spots, and ezogabine treatment of 11 patients. *Neurol. Genet.* **2**, e96. 10.1212/NXG.0000000000000096 (2016). 27602407 10.1212/NXG.0000000000000096 PMC4995058

[22] Singh, N. A. et al. A novel potassium channel gene, KCNQ2, is mutated in an inherited epilepsy of newborns. *Nat. Genet.* **18**, 25–29 (1998). 9425895 10.1038/ng0198-25

[23] Charlier, C. et al. A pore mutation in a novel KQT-like potassium channel gene in an idiopathic epilepsy family. *Nat. Genet.* **18**, 53–55 (1998). 9425900 10.1038/ng0198-53

[24] Singh, N. A., Otto, J. F., Dahle, J., Pappas, E. & Leslie, C. Mouse models of human KCNQ2 and KCNQ3 mutations for benign familial neonatal convulsions show seizures and neuronal plasticity without synaptic reorganization. *J. physiol.* **586**, 3405–3423 (2008). 18483067 10.1113/jphysiol.2008.154971 PMC2538806

[25] Maljevic, S. & Lerche, H. Potassium channel genes and benign familial neonatal epilepsy. *Prog Brain Res.* **213**, 17–53 (2014). 25194482 10.1016/B978-0-444-63326-2.00002-8

[26] Uchida, T. et al. Abnormal γ-aminobutyric acid neurotransmission in a Kcnq2 model of early onset epilepsy. *Epilepsia* **58**, 1430–1439 (2017). 28575529 10.1111/epi.13807

[27] Hinojo-Perez, A. et al. The conductance of KCNQ2 and its pathogenic variants is determined by individual subunit gating. *Sci. Adv.* **11**, eadr7012. 10.1126/sciadv.adr7012 (2025). 40043113 10.1126/sciadv.adr7012 PMC11881901

[28] Jensen, M. Ø. et al. Mechanism of voltage gating in potassium channels. *Science* **336**, 229–233 (2012). 22499946 10.1126/science.1216533

[29] Brown, M. S., Ye, J., Rawson, R. B. & Goldstein, J. L. Regulated intramembrane proteolysis: A control mechanism conserved from bacteria to humans. *Cell* **100**, 391–398 (2000). 10693756 10.1016/s0092-8674(00)80675-3

[30] Morohashy, Y. & Tomita, T. Protein trafficking and maturation regulate intramembrane proteolysis. *Biochim. Biophys. Acta*. **1828**, 2855–2861 (2013). 23770323 10.1016/j.bbamem.2013.06.001

[31] Wolfe, M. S. & Kopan, R. Intramembrane proteolysis: Theme and variations. *Science* **305**, 1119–1123 (2004). 15326347 10.1126/science.1096187

[32] Urban, S. & Freeman, M. Intramembrane proteolysis controls diverse signalling pathways throughout evolution. *Curr. Opin. Genet. Dev.* **12**, 512–518 (2002). 12200155 10.1016/s0959-437x(02)00334-9

[33] Holtzman, D. M., Morris, J. C. & Goate, A. M. Alzheimer’s disease: The challenge of the second century. *Sci. Transl Med.* **3**, 77sr1. 10.1126/scitranslmed.3002369 (2011). 21471435 10.1126/scitranslmed.3002369 PMC3130546

[34] Iwatsubo, T. et al. Visualization of Aβ42 (43) and Aβ40 in senile plaques with end-specific Aβ monoclonals: Evidence that an initially deposited species is Aβ42 (43). *Neuron* **13**, 45–53 (1994). 8043280 10.1016/0896-6273(94)90458-8

[35] Kanehara, K., Ito, K. & Akiyama, Y. YaeL (EcfE) activates the ςE pathway of stress response through a site-2 cleavage of anti-ςE, RseA. *Genes Dev.* **16**, 2147–2155 (2002). 12183368 10.1101/gad.1002302 PMC186437

[36] Strisovsky, K. Why cells need intramembrane proteases–a mechanistic perspective. *FEBS J.* **283**, 1837–1845 (2016). 26716760 10.1111/febs.13638

