# Supplementary material for: Novel proteolytic post-translational modification in voltage-gated potassium channel KCNQ2
Source: Sci Rep. 2026 Mar 4;16:11954. doi: 10.1038/s41598-026-42444-9 (PMC13069109; doi:10.1038/s41598-026-42444-9)
Supplement: Supplementary file 1 — Supplementary Material 1 [file 41598_2026_42444_MOESM1_ESM.pdf]

Novel proteolytic post-translational modification in voltage-gated potassium channel KCNQ2

Yuichi Kimura[1]*, Hidehiko Uchiyama[1], Koji Masuda[1], and Shinichi Hirose[2]

[1] Department of Animal Science, Tokyo University of Agriculture, Kanagawa, Japan.
[2] General Medical Research Center, School of Medicine, Fukuoka University, Fukuoka, Japan.

*Corresponding author
e-mail: yk203121@nodai.ac.jp

Supplementary Figure 1

Myc tag ↱ mKCNQ2

1 MEQKLISEEDLLMAMEARIRSTMVQKSRNGGVYPGTSGEKKLKVGFVGLD 50

51 PGAPDSTRDGALLIAGSEAPKRGSVLSKPRTGGAGAGKPPKRNAFYRKLQ 100

S1 S2

101 NFLYNVLERPRGWAFIYHAYVFLLVFSCLVLSVFSTIKEYEKSSEGALYI 150

S3

151 LEIVTIVVFGVEYFVRIWAAGCCCRYRGWRGRLKFARKPFCVIDIMVLIA 200

S4

201 SIAVLAAGSQGNVFATSALRSLRFLQILRMIRMDRRGGTWKLLGSVVYAH 250

S5

251 SKELVTAWYIGFLCLILASFLVYLAEKGENDHFDTYADALWWGLITLTTI 300

S6

301 GYGDKYPQTWNGRLLAATFTLIGVSFFALPAGILGSGFALKVQEQHRQKH 350

351 FEKRRNPAAGLIQSAWRFYATNLSRTDLHSTWQYYERTVTVPMYRLIPPL 400

401 NQLELLRNLKSKSGLTFRKEPQPEPSPSPRGMAAKGKGSPQAQTVRRSPS 450

451 ADQSLDDSPSKVPKSWSFGDRSRTRQAFRIKGAASRQNSEEASLPGEDIV 500

501 EDNKSCNCEFVTEDLTPGLKVSIRAVCVMRFLVSKRKFKESLRPYDVMDV 550

551 IEQYSAGHLDMLSRIKSLQSRIDMIVGPPPPSTPRHKKYPTKGPTAPSRE 600

601 SPQYSPRVDQIVGRGPTITDKDRTKGPAETELPEDPSMMGRLGKVEKQVL 650

651 SMEKKLDFLVSIYTQRMGIPPAETEAYFGAKEPEPAPPYHSPEDSRDHAD 700

701 KHGCIIKIVRSTSSTGQRNYAAPPAIPPAQCPPSTSWQQSHQRHGTSPVG 750

mKCNQ2 ↰ 3 x FLAG tag

751 DHGSLVLRLERSAGMMSCHLEVLFQGPGSGSTMDYKDHDGDYKDHDIDYK 800

801 DDDDK Stop 805

Supplementary Fig. 1. Full length amino acid sequence of mKCNQ2 with epitope tag.

Supplementary Figure 2

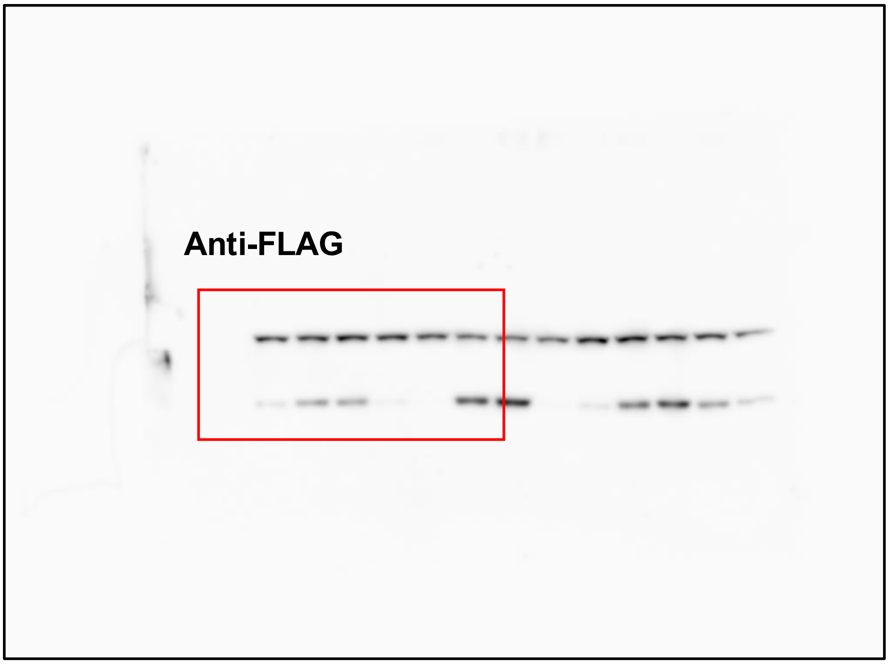

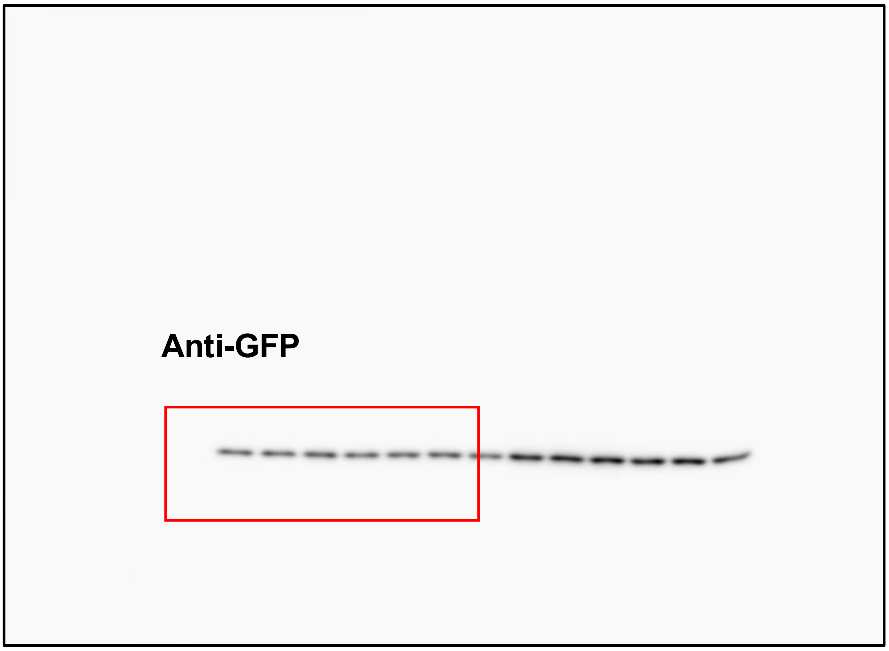

Supplementary Fig. 2. Original data for Fig. 1B

Supplementary Figure 3

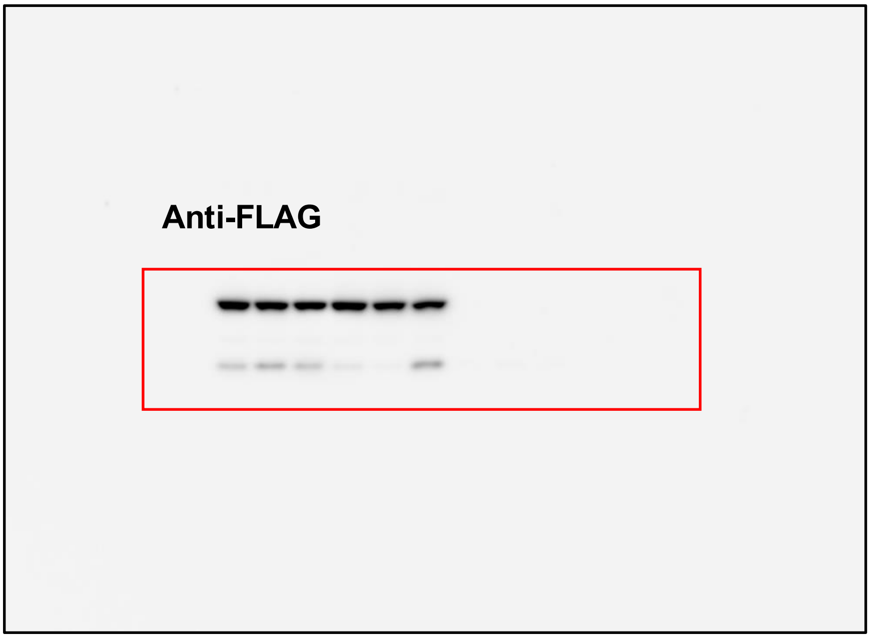

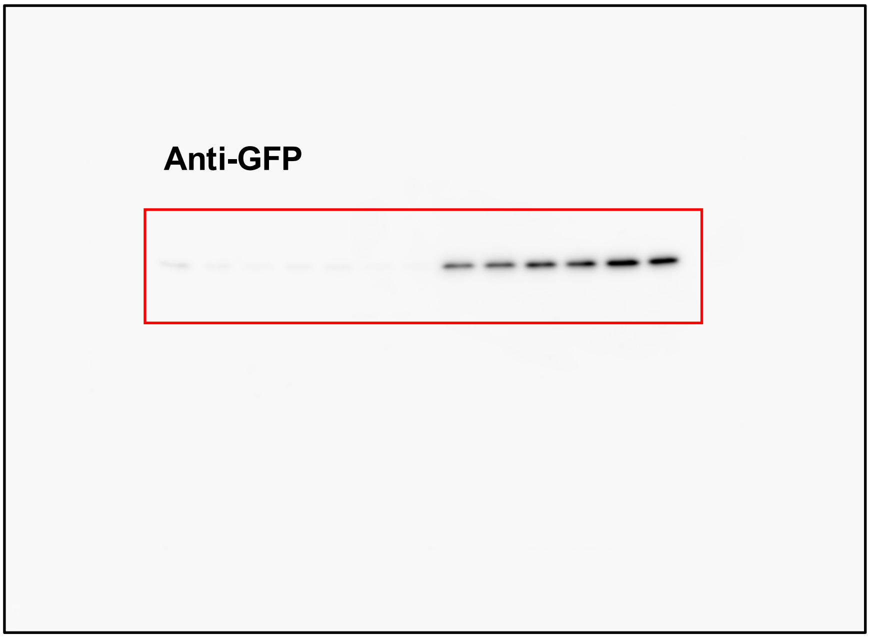

Supplementary Fig. 3. Original data for Fig. 2A

Supplementary Figure 4

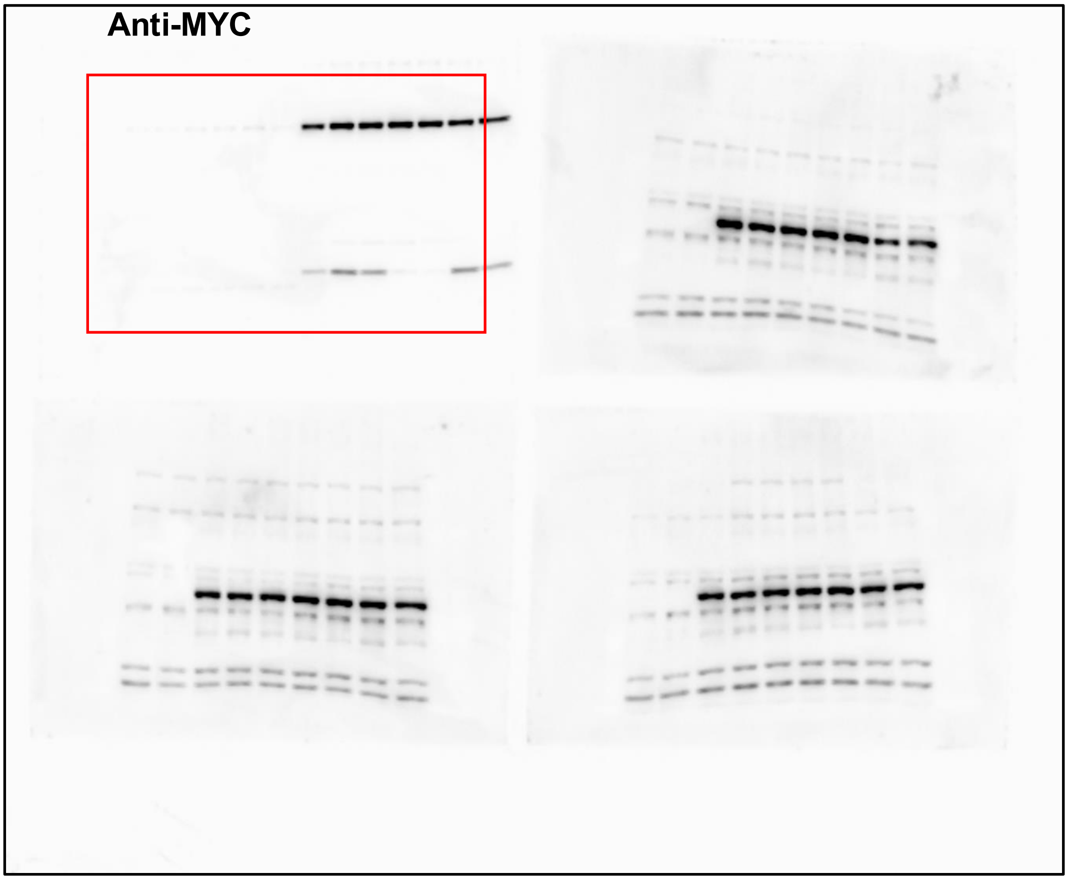

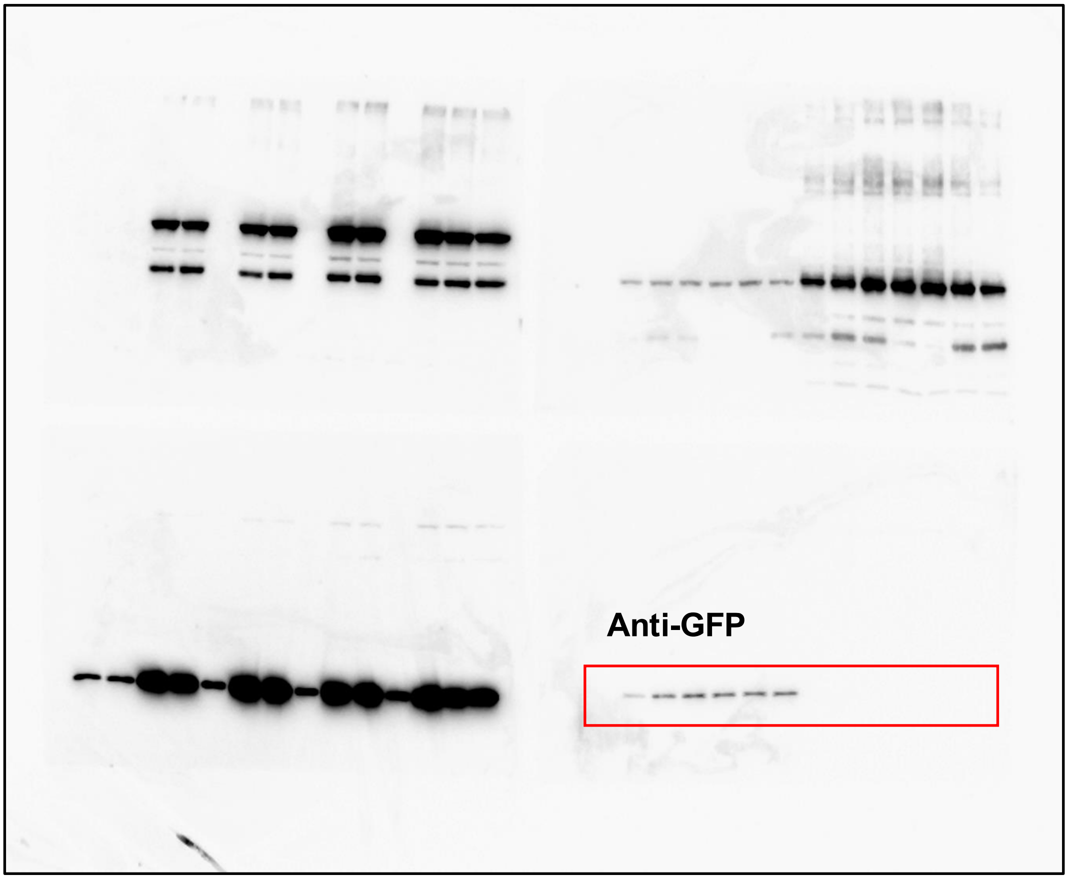

Supplementary Fig. 4. Original data for Fig. 2C

Supplementary Figure 5

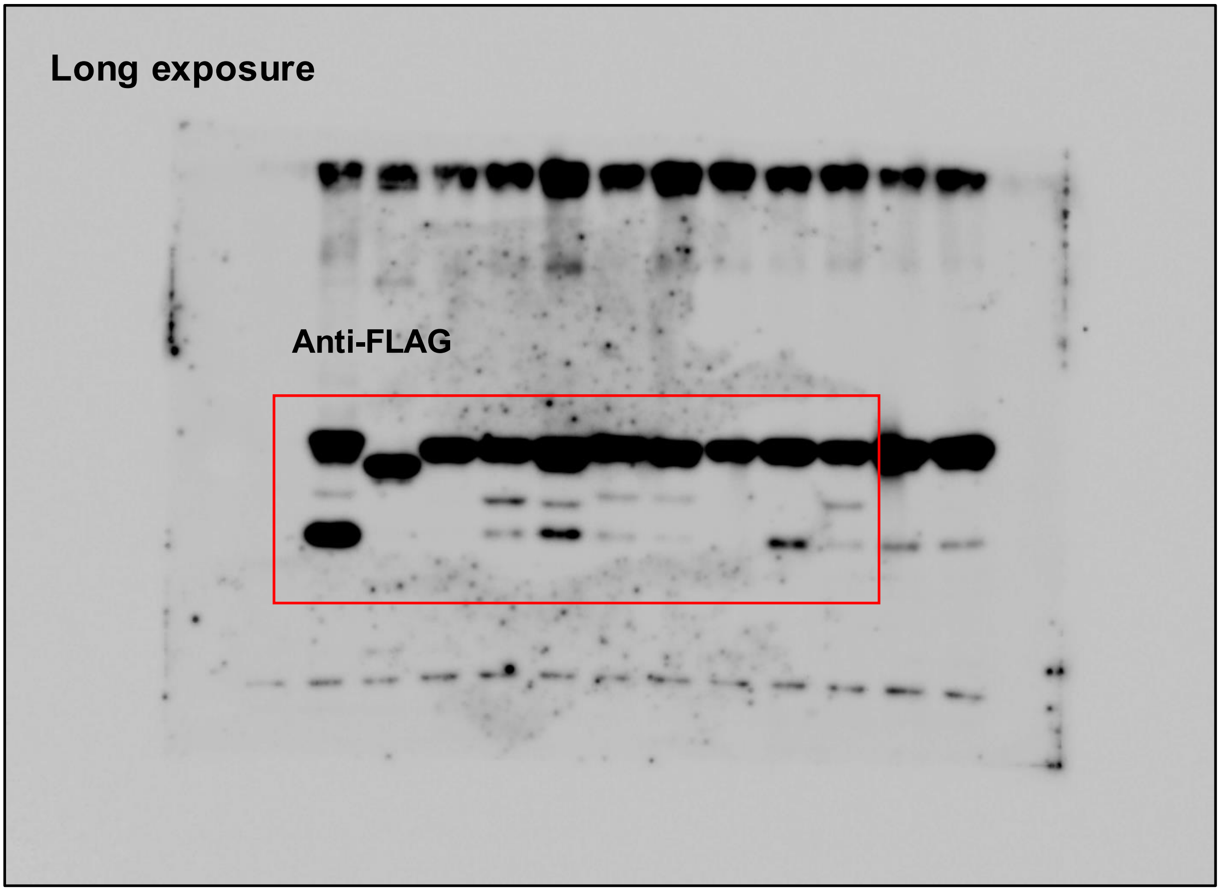

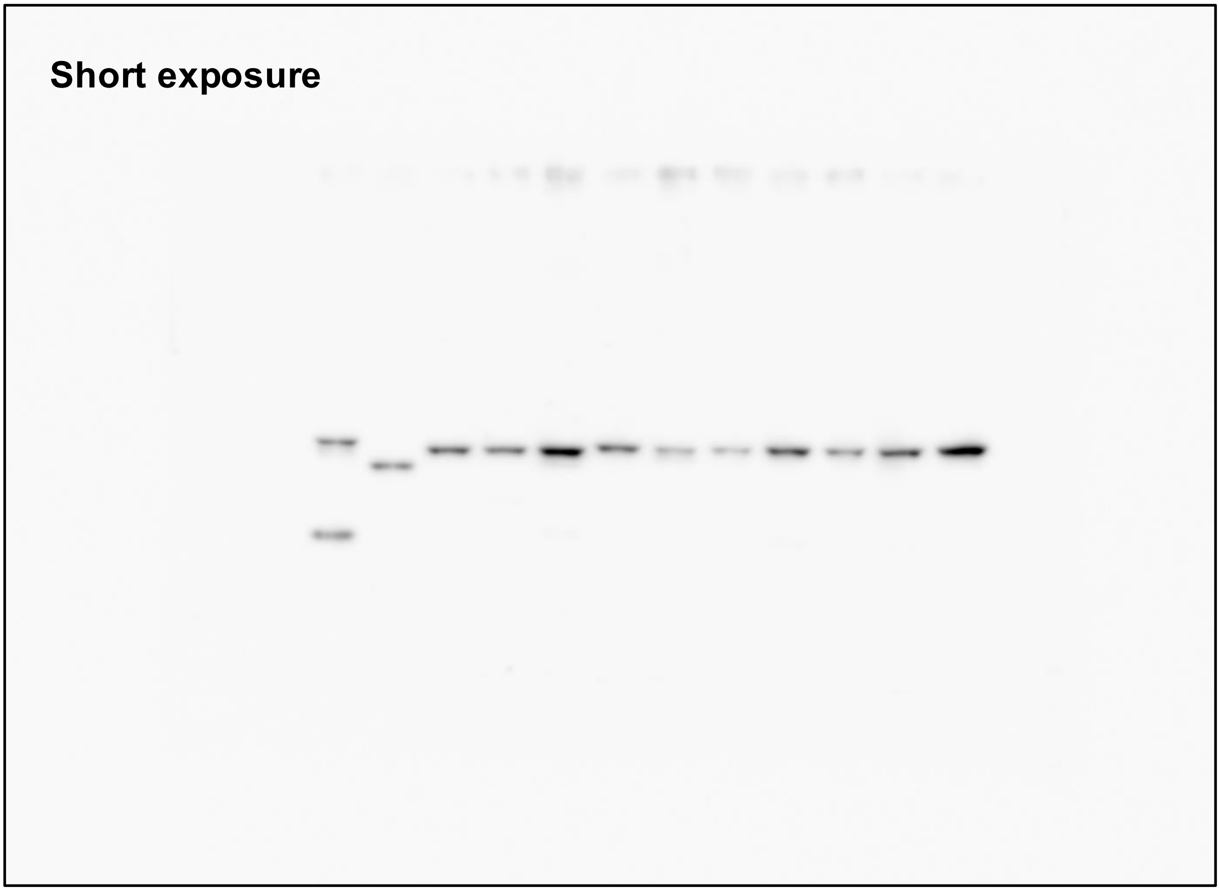

Supplementary Fig. 5. Original data for Fig. 3B

Supplementary Figure 6

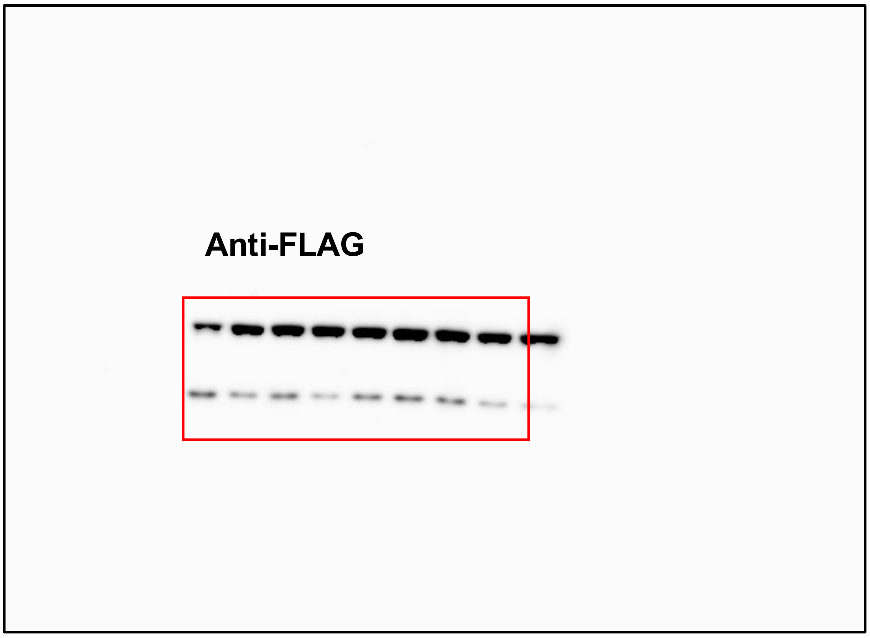

Supplementary Fig. 6. Original data for Fig. 3C

Supplementary Figure 7

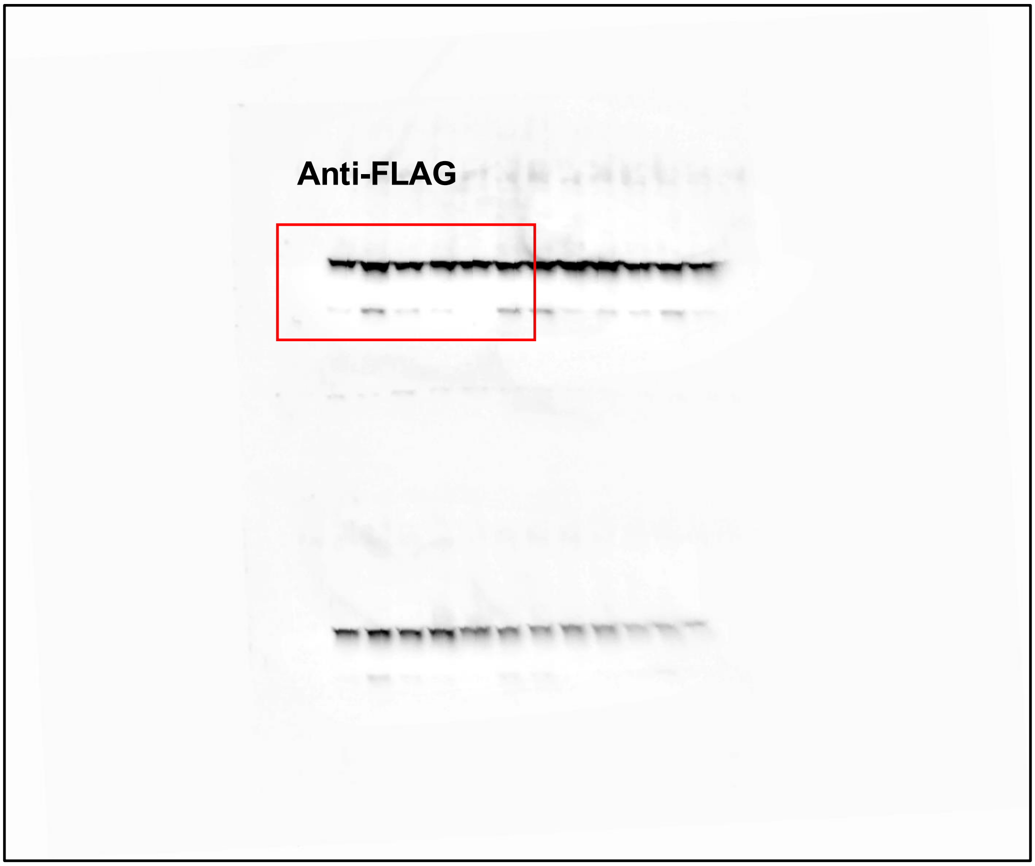

Supplementary Fig. 7. Original data for Fig. 3D

Supplementary Figure 8

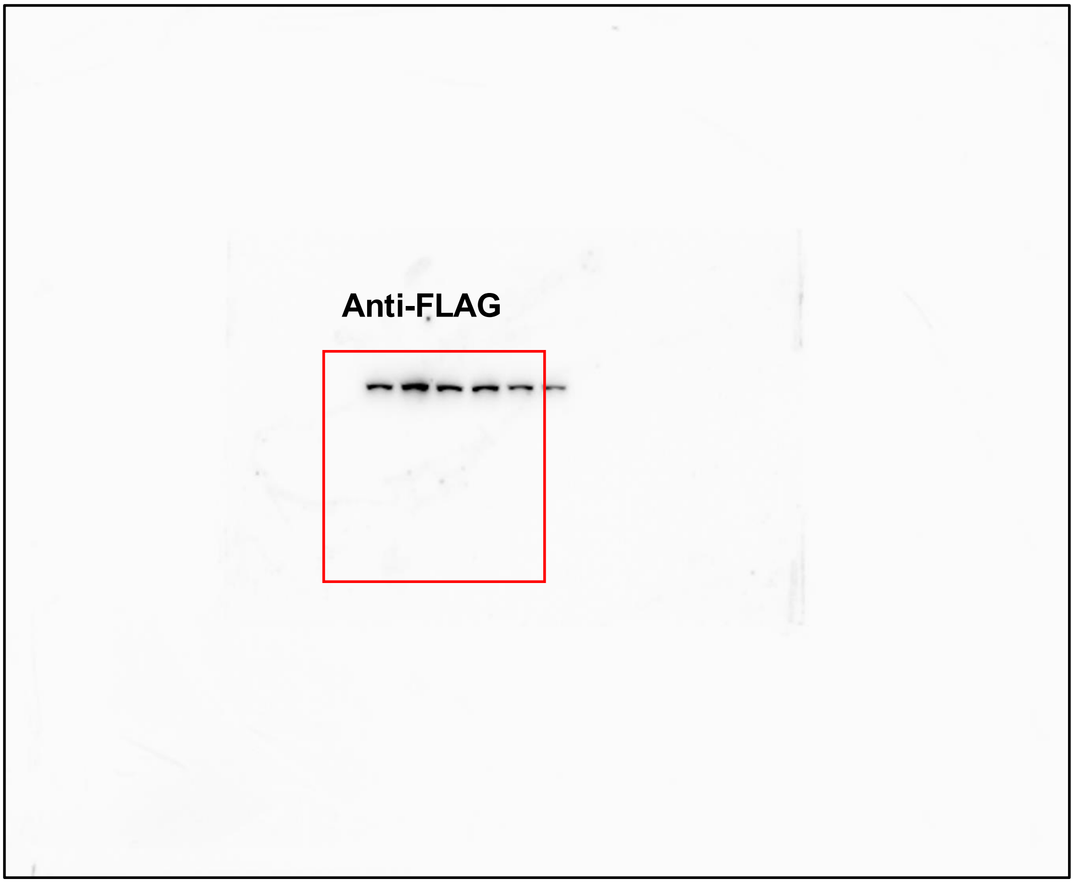

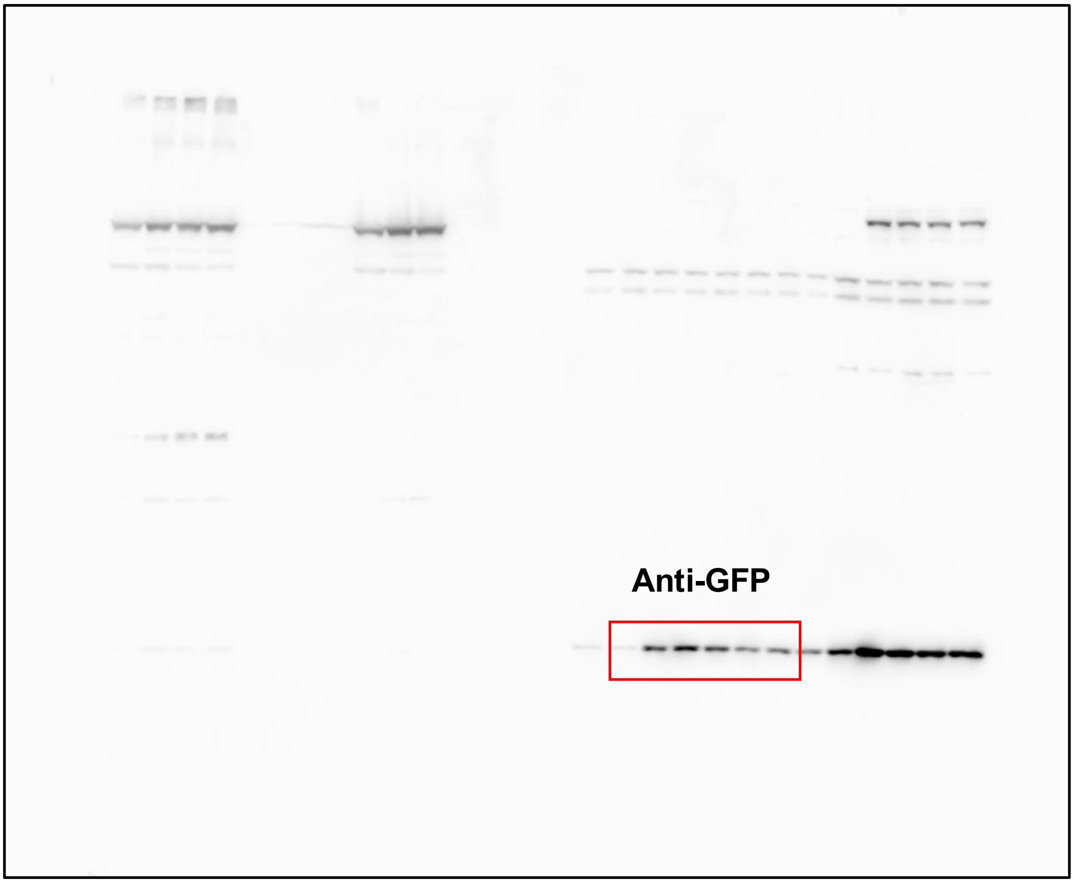

Supplementary Fig. 8. Original data for Fig. 4B

Supplementary Figure 9

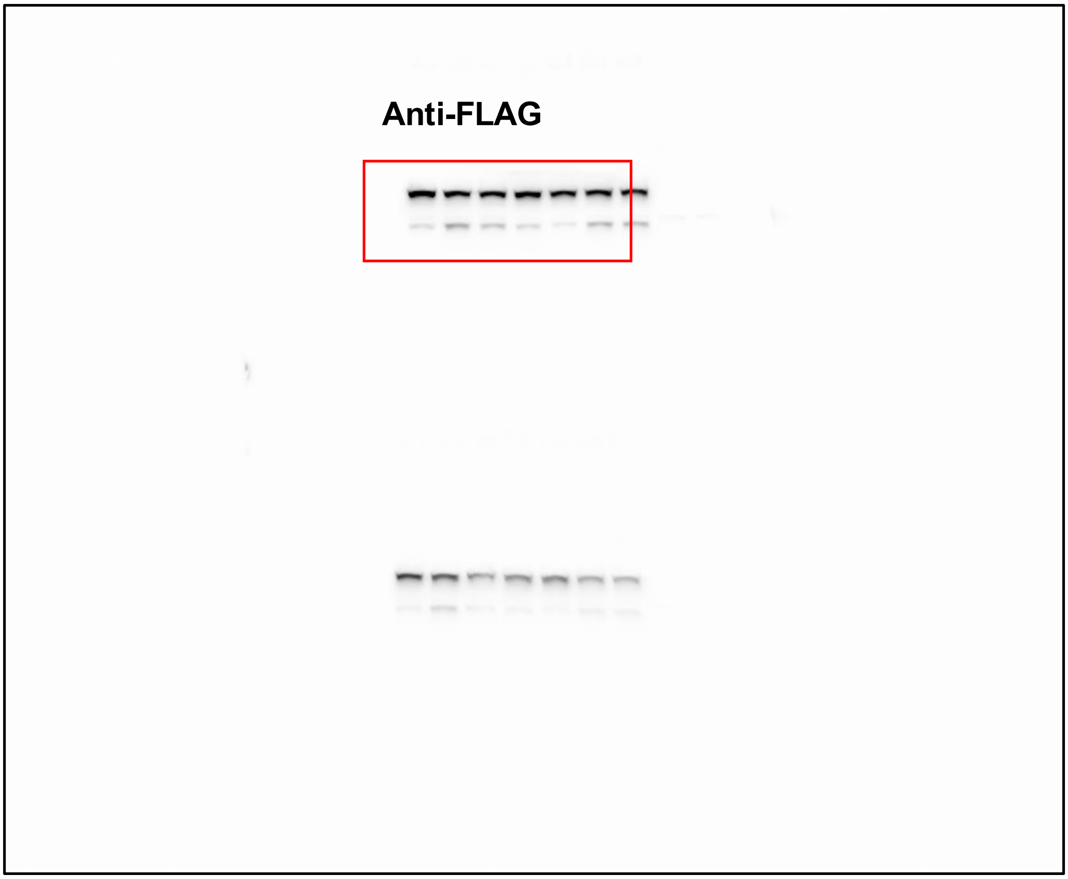

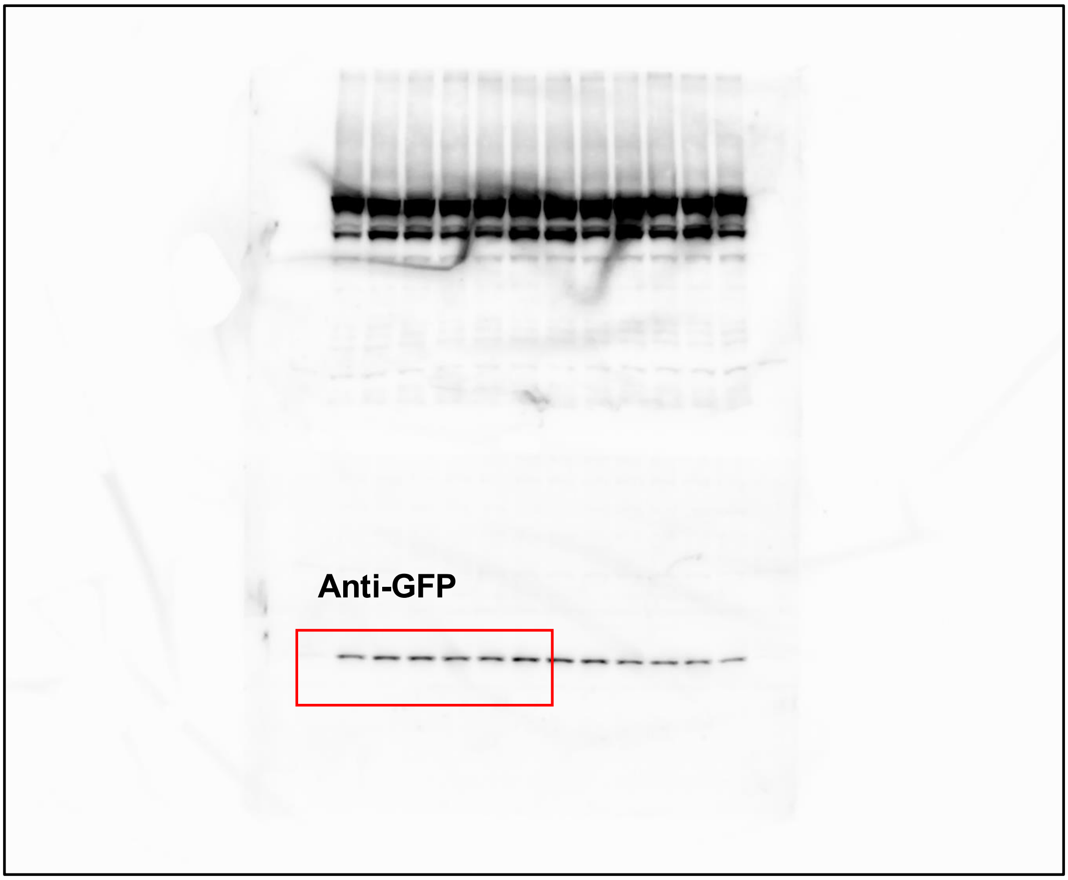

Supplementary Fig. 9. Original data for Fig. 5B

Supplementary Figure 10

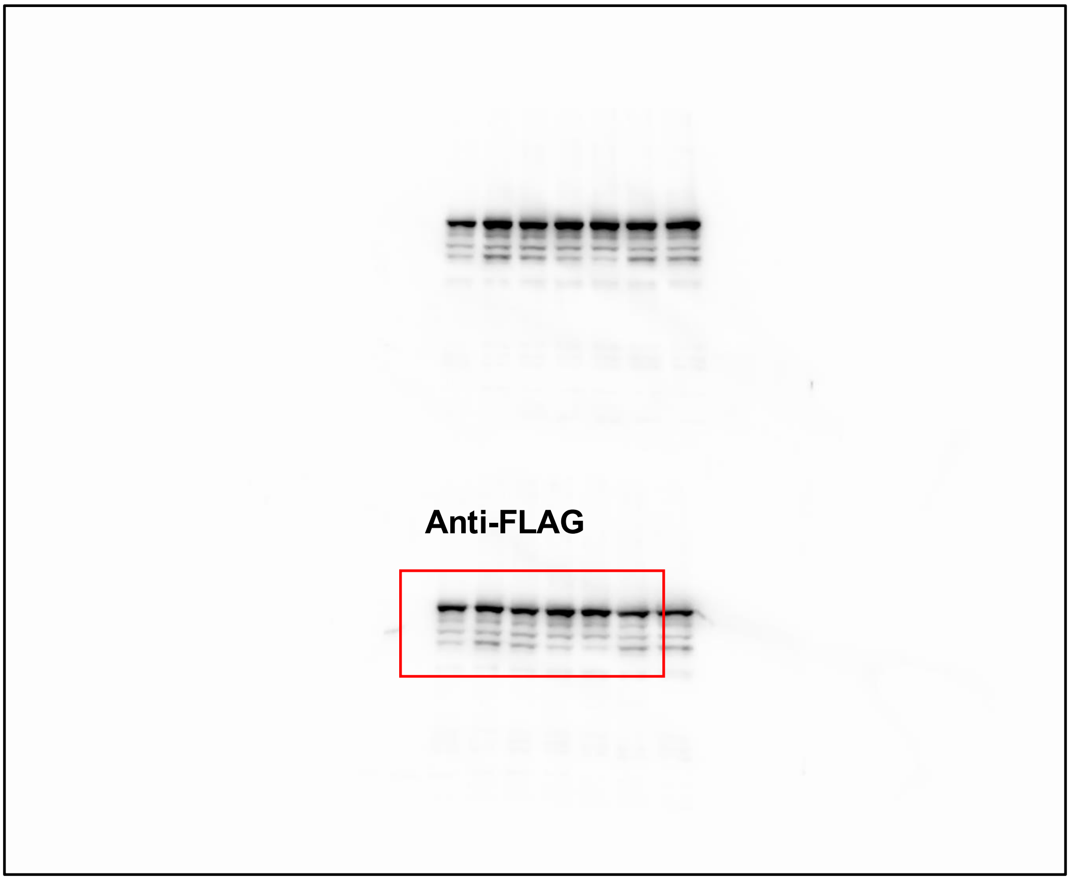

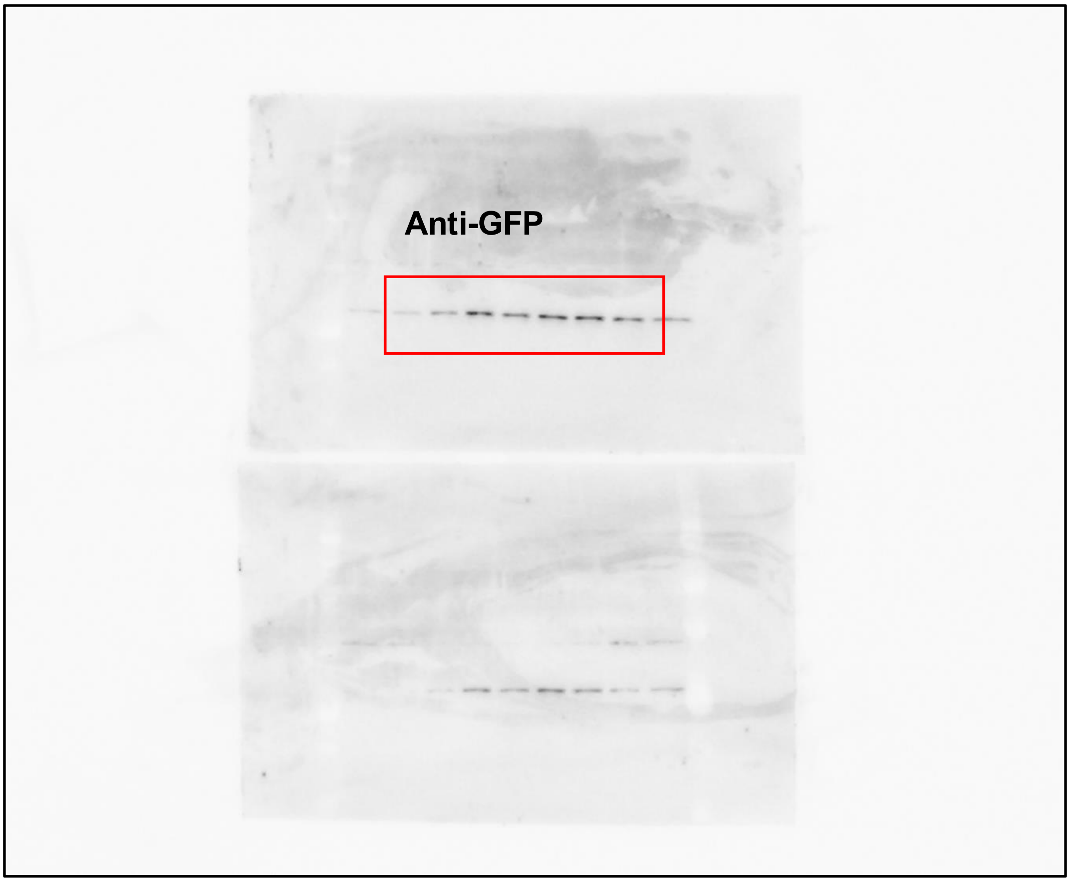

Supplementary Fig. 10. Original data for Fig. 5D

Supplementary Figure 11

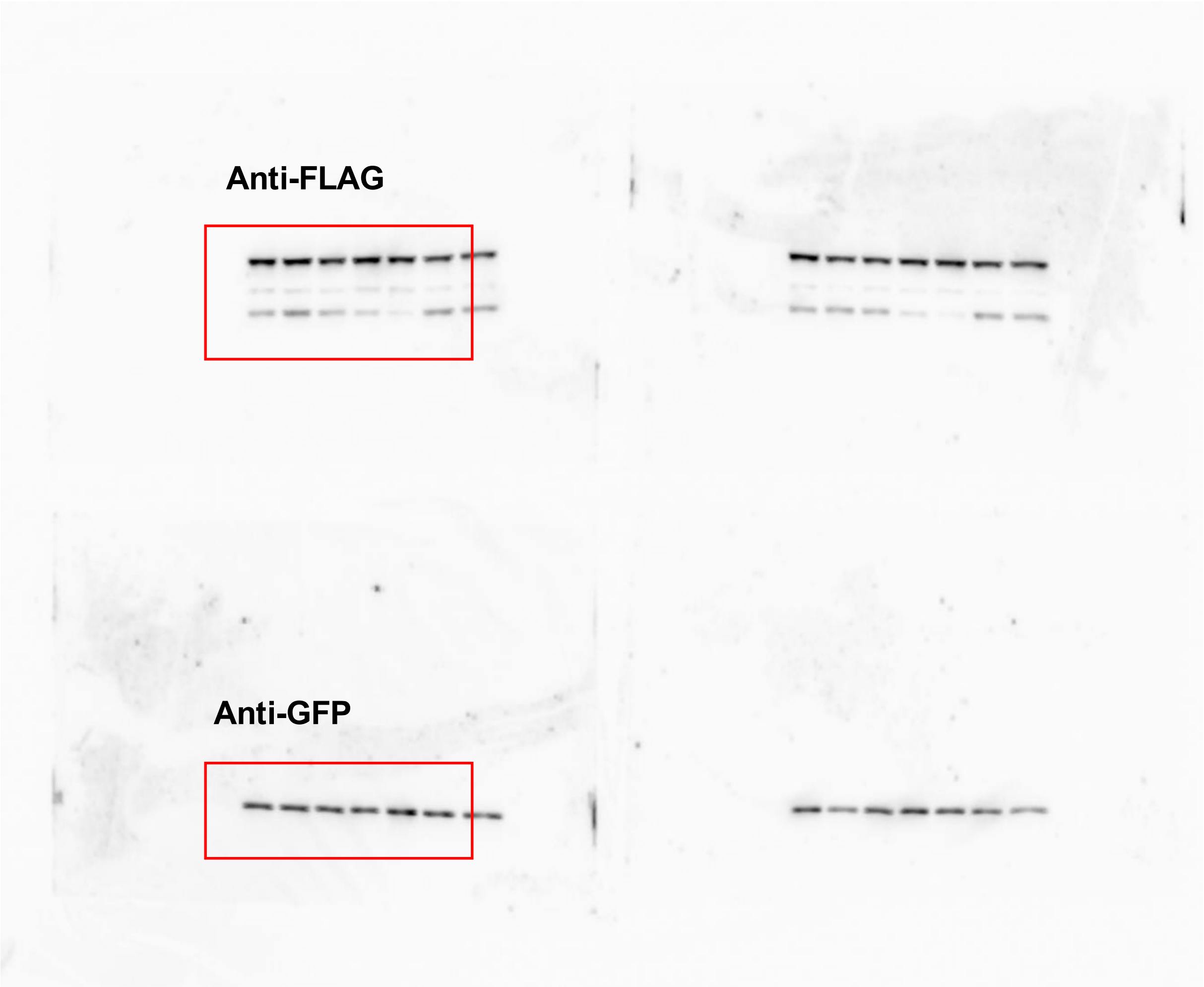

Supplementary Fig. 11. Original data for Fig. 5F
